# Characterization and expression patterns of *let-7 *microRNA in the silkworm (*Bombyx mori*)

**DOI:** 10.1186/1471-213X-7-88

**Published:** 2007-07-25

**Authors:** Shiping Liu, Qingyou Xia, Ping Zhao, Tingcai Cheng, Kaili Hong, Zhonghuai Xiang

**Affiliations:** 1 The Key Sericultural Laboratory of Agricultural Ministry, College of Biotechnology, Southwest University, Chongqing 400716, China

## Abstract

**Background:**

*lin-4 *and *let-7*, the two founding members of heterochronic microRNA genes, are firstly confirmed in *Caenorhabditis elegans *to control the proper timing of developmental programs in a heterochronic pathway. *let-7 *has been thought to trigger the onset of adulthood across animal phyla. Ecdysone and *Broad-Complex *are required for the temporal expression of *let-7 *in *Drosophila melanogaster*. For a better understanding of the conservation and functions of *let-7*, we seek to explore how it is expressed in the silkworm (*Bombyx mori*).

**Results:**

One member of *let-7 *family has been identified in silkworm computationally and experimentally. All known members of this family share the same nucleotides at ten positions within the mature sequences. Sequence logo and phylogenetic tree show that they are not only conserved but diversify to some extent among some species. The *bmo-let-7 *was very lowly expressed in ova harvested from newborn unmated female adult and in individuals from the first molt to the early third instar, highly expressed after the third molt, and the most abundant expression was observed after mounting, particularly after pupation. The expression levels were higher at the end of each instar and at the beginning of each molt than at other periods, coinciding with the pulse of ecdysone and *BR-C *as a whole. Using cultured ovary cell line, BmN-SWU1, we examined the effect of altered ecdysone levels on *bmo-let-7 *expression. The expression was also detected in various tissues of day 3 of the fifth instar and of from day 7 of the fifth to pupa, suggesting a wide distributing pattern with various signal intensities.

**Conclusion:**

*bmo-let-7 *is stage- and tissue-specifically expressed in the silkworm. Although no signals were detected during embryonic development and first larval instar stages, the expression of *bmo-let-7 *was observed from the first molt, suggesting that it might also function at early larval stage of the silkworm. The detailed expression profiles in the whole life cycle and cultured cell line of silkworm showed a clear association with ecdysone pulse and a variety of biological processes.

## Background

The life rhythmicities in *Caenorhabditis elegans *and *Drosophila melanogaster *result from programmed mediation of a series of heterchronic genes [1-4]. Two small noncoding RNAs, *lin-4 *and *let-7*, are essential components of the heterochronic pathway dictating temporal decisions of cell fate from one stage to the next [4].*lin-4 *has long been thought to regulate the transition from the first to the second larval stages in *C. elegans *[5-7], and *let-7 *was also confirmed to be undetectable until the last larval stage and functions as a temporal switch promoting the transition from larval to adult stages [8,9]. However, the latest studies showed that *lin-4 *and one of its target genes, *lin-14*, also regulate life span in the adult of *C. elegans *[10], and that *let-7 *can even be found in the 3^rd ^instar larvae of *C.elegans *[11,12], and may be a master temporal regulator of late larval development in *C. elegans *[13]. In *Drosophila*,*let-7 *RNA first appears at the end of the third larval instar, a few hours before puparium formation, and reaches high levels during pupal development stage [9,14]. Both the temporal RNAs are functional in a mechanism through post-transcriptionally or translationally repressing their target genes. *lin-4 *inhibits the translations of *lin-14 *and *lin-28 *by base-pairing to partially complementary sites in the 3'-UTRs of their mRNAs [15,16], and *let-7 *has been confirmed to inhibit *lin-41*'s expression in a similar fashion through binding to complementary sites in its 3'UTR [17]. *let-7 *RNA is consistently detectable in samples from diverse animal phyla, including chordates, hemichordates, echinoderms, mollusks, annelids, and arthropods, but cannot be found in basal metazoans, such as cnidarians and poriferans, as well as in plants and unicellular organisms [8,18]. The diverse expression patterns of *let-7 *family in various species may be responsible for its important role in developmental regulation [19-21].

The expression level of temporal microRNAs was confirmed to respond to the pulse of steroid hormone 20-hydroxyecdysone (Ecd) and/or juvenile hormone (JH) [22]. Ecd and JH control the development of *Drosophila *from embryogensis to adulthood [23-25]. They exert the opposite effects on the expression of temporal miRNA genes, such as *mir-34*, *mir-100*, *mir-125 *and *let-7 *[22]. Furthermore, the opposite effects of JH and Ecd signals could be mediated by *Broad-Complex *(*BR-C*) [22]. The major developmental transitions in the life cycle of *Drosophila *are directed by the pulses of ecdysone [26,27], and ecdysone signals the stage-specific programmed cell death (PCD) of the larval salivary glands during *Drosophila *metamorphosis [28], and also triggers the programmed cell death in anterior silk glands (ASGs) of *Bombyx mori *[29]. Early Ecd-inducible genes,*E74, E75 *and *BR-C*, coordinate with the temporal and spatial activation of downstream genes, initiating a genetic cascade that leads to distinct metamorphic processes such as pupation, PCD of larval tissues, remodeling of the central nervous system, and proliferation and morphogenesis of imaginal discs [22,26,30]. Ecdysone and the Ecd-inducible gene *BR-C *are required in the upregulation of some microRNAs including *let-7 *as well as the downregulation of other microRNAs [22].

Although a bevy of *let-7 *family members exist in various organisms, their dos-a-dos in *B. mori *has not been described experimentally [31]. *B. mori *is a typical member of the family Bombycidae with about 300 moth species in the order Lepidoptera. It has recently become a model for studying the harmful Lepidopteran insects and the commercial production of useful biological substances called interleukins [32,33], especially after the completion of draft sequence of domesticated silkworm genome [34,35]. The overt roles of *lin-4 *and *let-7 *in switching the transition of metamorphosis in other organisms raise the question of whether similar regulatory RNAs are involved in the control of metamorphosis development of *B. mori *and how the temporal identity conferred by the heterochronic genes is related to the major developmental landmarks that define the silkworm life cycle. As a step towards a better understanding of roles of *let-7*, we seek to explore whether they exist in silkworm genome and how they are expressed during the life cycle of this insect.

## Results and discussion

### One homology of *let-7 *in the silkworm genome

**Figure 1:**
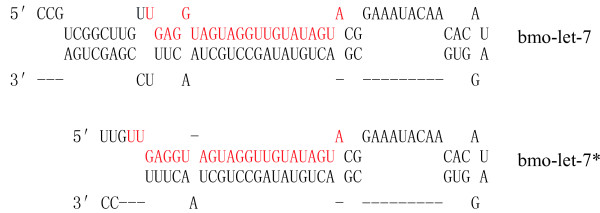
**Stem-loop structures of alternative forms of bmo-let-7 precursor**. The mature sequences are in red, shown in the foldback of the precursors.*let-7 *small RNA of silkworm is 22 nt or 23 nt long, and the precursor is 87 nt or 72 nt long. The actual size of the stem-loop structure is not known experimentally and may be slightly shorter or longer than represented.

By using of BLASTN based on sequence homology, we identified one orthologue of *let-7 *in the silkworm genome, which was named *bmo-let-7 *according to a common criterion (*Bombyx mori*, *bmo-*) [31]. The same site on the genome sequence of AADK01002497(NCBI) was further confirmed by using the miRscan program combined with RNAfold. The mature sequence and its precursor are at 10858–10879 and 10846–10932, respectively, or at 10857–10879 and 10854–10925, respectively. The mature sequence, either bmo-let-7 or bmo-let-7*, is on the 5' arm of the foldback precursor (Fig. 1). The potential alternative forms of this homology were submitted to Northern blotting analysis later in order to see if stable results could be obtained. Using the same prediction methods, however, we did not find *cel-lin-4 *homologue in the silkworm genome.

### The extending *let-7 *family shares common "miRNA seed" and represents restricted species diversities

**Figure 2:**
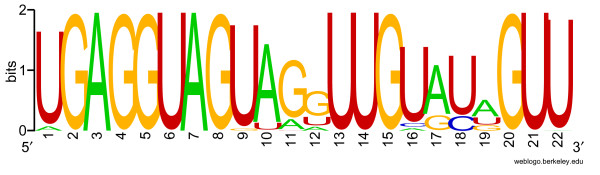
**Sequence logo of known *let-7 *family members**. Ninety known *let-7 *members together with bmo-let-7 were aligned using the CLUSTAL × program, then were submitted to logo analysis by using the WebLogo program available [66]. The height of the letters indicates the relative frequency of the letter at that position and the overall height of the stack indicates the sequence conservation in terms of information content in bits.

There have been over 90 members so far in *let-7 *family that contains about 30 different mature sequences, ranging in length from 19 nt to 22 nt. All members share the two common fragments, GAGGUAG and UUG (Fig. 2), and they might be the common "miRNA seed" which could be used to search for target genes according to the complementarity to sequences in the 3' UTR of all expressed genes [36]. Seven of ten shared positions are located at the 5' end of the mature sequence, and the other three at the 3' end, consistent with the proposal that the 5' end of the functional small RNA is crucial for the stability and proper loading of *let-7 *into the miRISC complex though both ends are required for downregulation of a target gene [37].

**Figure 3:**
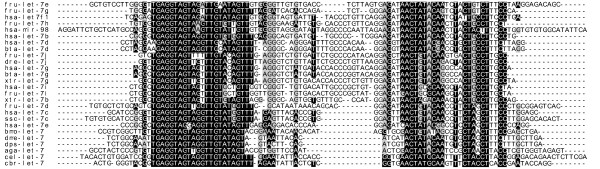
**Sample clusters of known precursors of *let-7 *family members based on sequence similarity**. Twenty-six of all known let-7 members were selected in terms of sequence- and species-specific and aligned against each other by a Smith-Waterman algorithm.

Twelve positions exhibit exchangeable if the mature seqence of *cel-let-7*, 5'-UGAGGUAGUAGGUUGUAUAGUU-3' (MIMAT0000001), is used as the reference, U at 5' end being position 1. For example, it is A at position 1 in five *let-7d *members, G at position 10 in three *let-7e *members, and U at position 10 in three other *let-7d *members. At position 11, G is replaced by A in three *let-7h *and eleven miR-98 members, or by U in both *fru-let-7j *and *tni-let-7j*. Position 12 is shared by three nucleotides, G, U and A in the mature sequences of all members. Four nucleotides near the 3' end, namely, positions 16 to 19 are also replaceable. Compared with the reference sequence (*cel-let-7*), *bmo-let-7 *substitutes the nucleotide U with A at 3' end, as an exception to uridine preference at both ends. In addition, deletion of nucleotides can take place at the 3'end. For example, *ssc-let-7 *is three nucleotides shorter than the control sequence and twenty-two members are one nucleotide shorter than the norm sequence (Figs. 2, 3). Taken together, the divergent sequences and replaceable nucleotides may imply their function diversities among species to some extent.

### The extending *let-7 *family might have evolved from a common ancient precursor

**Figure 4:**
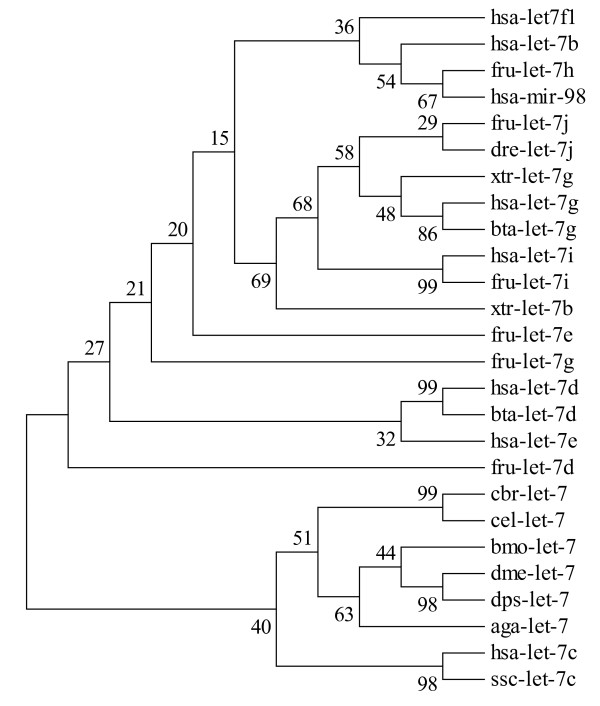
**Phylogenetic tree based on the selected precursors of let-7 family members**. In order to be convenient for analysis, we selected 26 representative let-7 precursors. Phylogenetic tree was constructed using MEGA v3.0 with 1000 times bootstrap sampling. Full species names coresponding to the microRNAs are as follows: hsa, *Homo sapiens*; fru, *Fugu rubripes*; dre, *Danio rerio*; xtr, *Xenopus tropicalis*; bta, *Bos taurus*; cbr, *Caenorhabditis briggsae*; cel, *Caenorhabditis elegans*; bmo, *Bombyx mori; *dme, *Drosophila melanogaster*; dps, *Drosophila pseudoobscura*; aga, *Anopheles gambiae*; ssc, *Sus scrofa*; tni, *Tetraodon nigroviridis*.

*C. briggsae*, *C. elegans*, *B. mori*, *D. melanogaster*, *D. pseudoobscura *and *A. gambiae *are Ecdysozoa, Protostomia, and all *let-7 *precursors of them are clustered in the same clade (Fig. 4). *B. mori *belongs to Insecta, Arthropoda, and *bmo-let-7 *precursor is clustered with those of other insects, accordingly. *Homo sapiens*, *Fugu rubripes*, *Danio rerio*, *Xenopus tropicalis*, *Bos taurus *belong to Chordata Deuterostomia, and *let-7 *precursors of them, except for *has-let-7 *and *ssc-let-7*, are clustered in one clade. The conserved nucleotides of the precursors are those that tend to form the mature sequences. Every small RNA locates closely to the 5' end of its precursor. However, the loop regions are significantly more divergent than their mature sequences between mammalians and insects (Fig. 4). The conserved fragments on both arms of the precursors and the variations in loop regions support the hypothesis that an ancient precursor of the *let-7 *genes may have been common to the earliest animal lineages [18].

### It is detectable in the silkworm from the early first molt to the adult, suggesting that *bmo-let-7 *might even function in early larval stages of silkworm

**Figure 5:**
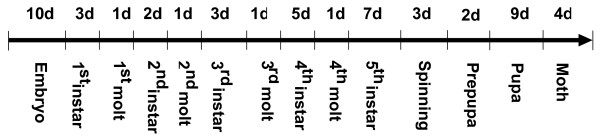
**Stage durations in the lifespan of *B. mori***. When reared at 25°C and 85% H.R., the lifespan of silkworm is over 50 days, consisting of ova, larva, pupa and imago. The embyo duration is about 10 days if proper acid-treatment is performed at hour 24 after oviposition. The first instar is 3 days long, starting at the newly-hatched silkworm or named ant silkworm, ending at just stopping eating mulberry leaves for the first molt. After spinning, the silkworm will live through pre-pupa of two days before becoming pupae. The durations vary a lot, ranging from 1 to 10 days.

**Figure 6:**
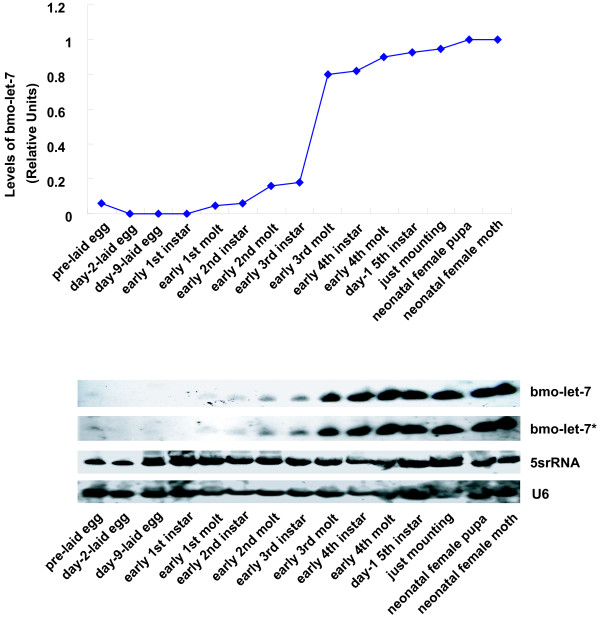
**General profile of *bmo-let-7 *accumulation during development of silkworm**. Both the sense and antisense probes were examined here, but only two antisense probes brought out the same positive results. And *lin-4 *escaped detection in all samples. All samples were quantified to be 15 μg per well before loading (see Materials and Methods). U6 and 5srRNA hybridizations were used as loading controls and levels of *let-7 *RNA are quantified relative to 5srRNA. Reproducible and consistent results were confirmed experiments repeated (data not shown).

**Figure 7:**
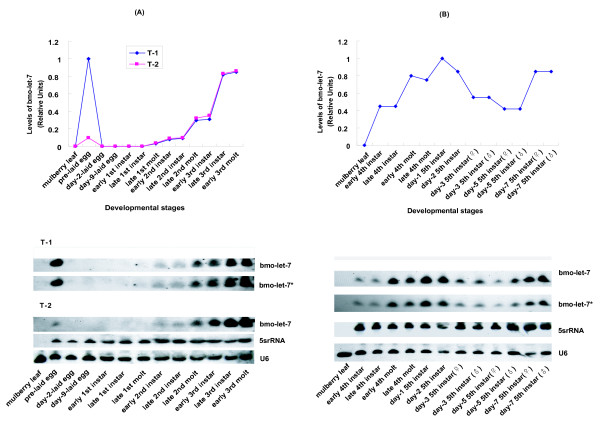
**Detailed profile of *bmo-let-7 *accumulation from ova to the late fifth instar**. (A). Expression profile from pre-laid egg (ova) to to early 3^rd ^molt. In the first test, the oviduct attached to egg was not discarded. In the second test, the oviduct was completely removed. T-1 stand for the first test (Test 1), T-2 means the second test (Test 2). The mulberry leaf was mainly used as a control to eliminate the potential contamination. U6 and 5srRNA hybridizations were used as loading controls and levels of *let-7 *RNA are quantified relative to 5srRNA. (B). Expression profile from early 4^th ^instar to day-7 5^th ^instar. The female and male silkworms were differentiated on the basis of gonad while harvesting the materials. U6 and 5srRNA hybridizations were used as loading controls and levels of *let-7 *RNA are quantified relative to U6.

*let-7 *is notably induced at the last larval stage in *C. elegans*, promoting the transformation from the larva to the adult [9,11], and highly expressed in the late third instar larvae of *D. melanogaster *when a pulse of the ecdysone triggers puparium formation and onset of metamorphosis [14]. In the case of *bmo-let-7*, we observed a wide expression profile by using of fifteen stage-specific samples across the lifespan of the silkworm (Figs. 5, 6). The expression of *bmo-let-7 *started at the early first molt, remained at low levels until the early third instar, and rapidly reached high level at the early third molt, still increased gradually to the maximum at pupa and imago. The whole larval duration from the first to the fifth instars could be divided into two parts with the boundary at the third instar on the basis of expression level of *bmo-let-7 *(Figs. 6 and 7), in accordance with the division determined by the pulse of ecdysone in the first four instars [38]. Approximately, the general dynamic changes in *bmo-let-7 *levels is also close to the pulse of *Broad-Complext *(*BR-C*) transcriptional factor of silkworm, which is hardly detectable before the fourth instar, lowly expressed in the fifth instar, and largely induced during cocooning and pupation when the ecdysone titer increases [39-41]. The coordinated changes imply that the developmental transitions of silkworm are more likely to be triggered by the cooperation of ecdysone, *BR-C*, *bmo-let-7 *and many other downstream genes. The wide-range emergence implies that *bmo-let-7 *not only controls the metamorphosis from late laval stage to the pupa, as do the other members in let-7 family confirmed in *C. elegans *[9,13] and *D. melanogaste*r [8,14], but might even function in the early larval stages of silkworm. The antisense probes of *bmo-let-7 *and bmo-let-7* brought out the same results in our sequential and parallel blotting experiments. The two sense probes, however, gave no signals in all samples, confirming the single strand of this small functional RNA. c*el-lin-4 *was beyond detection in the silkworm by Northern blotting with the antisense and sense probes, consistent with the prediction results.

**Figure 8:**
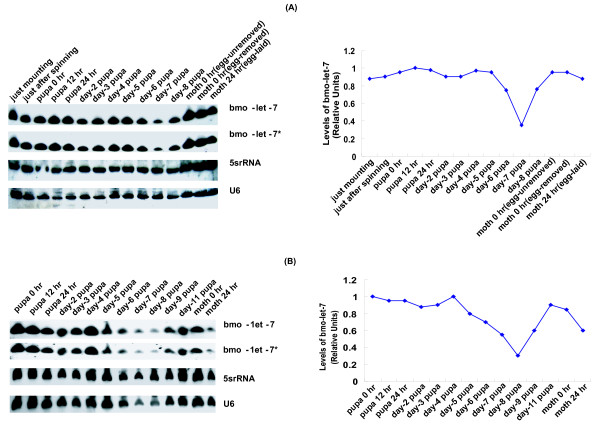
**Detailed expression profile of bmo-let-7 during *B.mori *pupa metamorphosis**. (A). Profiling during female pupa metamorphosis under artificial culture conditions. (B). Profiling during male pupa metamorphosis under natural culture conditions. The two groups were incubated under different conditions, group A under 25°C and 85%RH, group B under 18–30°C and 40–100%RH. Spinning stage, as well as adult stage, was simultaneously tested in favor of comparative analysis. Two or three days after mounting, cocoon spinning is finished and the larva becomes prepupa. In the following two or three days, the prepupa becomes pupa after molting. The pupa takes 8~11 days to transform into a moth which is white-grey colored. Under natural conditions(18~30°C), the pupa metamorphosis will take 10~11 days; Under man-made conditions (25°C), however, pupa lasts 8~9 days ended as adult moth. U6 and 5srRNA were used as loading controls. 5srRNA and U6 were used as the loading controls and levels of *bmo-let-7 *RNA are quantified relative to U6.

### Detailed expression profiles from unfertilized egg (ova) to the third molt and from the early fourth instar to the late fifth instar

After studying the general expression profile of *bmo-let-7 *across the whole lifespan (Fig. 5) with fifteen samples (Fig. 6), we further investigated its expression patterns at the turning point of each stage. As shown in the expression profiles from unfertilized egg (ova) to the adult and from ova to the third molt (Fig. 7A), as well as from the early fourth instar to day-7 in the fifth instar (Fig. 7B), the signal intensity at the beginning of each molt was stronger than that at the latest former early instar. For example, the signal intensities at the early second and third molts almost doubled those at the early second and third instars, respectively (Fig. 6); and the signal intensity at the early fourth molt was also higher than that at the early fourth instar (Fig. 7A). High level of ecdysone is required to induce some heterochronic genes before molt, and *let-7 *was also confirmed to be induced by pulse of ecdysone in *D. melanogaster *[22]. Both *let-7 *and ecdysone may be crucial for the transformation of larval stages [14]. During the development of silkworm, ecdysone peak comes several hours before the beginning of each molt, and a fall occurs at ecdysis [38], slightly ahead of scheduel of the expression profile peak of *bmo-let-7 *in our tests, well supporting the proposal that *bmo-let-7 *may be induced by the pulse of ecdysone.

It has been demonstrated that not conjugated but free ecdysone plays an important role in the control of cuticle formation in the embryo [42]. The unfertilized egg (ova) before oviposition removed from the female abdomen gave a weak signal of this microRNA (Figs. 6, 7A-T2), probably due to the slight induction by maternity genes or/and by large amount of free ecdysone in the adult organ, oviduct, which overly expressed *bmo-let-7 *(Fig. 7A-T1). *bmo-let-7 *was undetectable in diapause eggs (data not shown) because of very low free ecdysteroid fraction [42]. For the same reason, no signal was found in the embryonic development stages at day-2, day-6 and day-9 after oviposition at 25°C (Figs. 6, 7A), and in the first instar larval stages, even though the free ecdysteroid fraction is much larger than the conjugated ecdysteroid fraction in early developing eggs from the second to the fifth day[42,43]. Probably, it is not the conjugated ecdysteroid but the free ecdysone that functions in inducing the expression of *bmo-let-7 *and only when the amount of free ecdysone is over a threshold can function properly.

The signal intensity at the beginning of molt was slightly stronger than that at the end of molt. For example, the expression levels of *bmo-let-7 *were higher at the early first and fourth molts than those at the late first and fourth molts, respectively (Figs. 6, 7A, B). The instar stages, however, showed different expression profiles. The signal intensity at the beginning of the second and fourth instars was almost identical to that at the end of the second and fourth instars, respectively. A big jump in the expression profile was observed relative to at the beginning of third instar, further confirming the watershed between the third instar and fourth instar where comes the maximum peak of ecdysone [38]. The duration of the fifth instar is usually 7 days, and the expression pattern during this period was relatively broad. *bmo-let-7 *was highly expressed at day 1 and day 2, relatively lowly expressed at day 3 and day 5, then again highly expressed at the end of the fifth instar. Moreover, there was no expression difference between female and male during this stage (Fig. 7B). Taken together, each upsurge occurs at the turning point between developmental stages before maturing, suggesting that *bmo-let-7 *is required for the transformation of larval stages.

### A falling in the high level of expression profile is shared by female and male pupa metamorphosis at pupal-moth ecdysis

A strong signal was observed in the new female pupa and adult (Fig. 6). Northern blotting in this assay, however, revealed an extraordinarily high expression profile across the whole process of pupation and eclosion of females and males. A strong signal of the expression was observed at day-7 of the fifth instar when the silkworm is ripe for mounting or spinning cocoons (Fig. 7B). After spinning, the fifth instar larvae enter a prepupation stage. There was no expression difference between the beginning and the end of spinning stage, implying that *bmo-let-7 *is unlikely related to the process of spinning. The silkworm prepupa undergoes a molt before pupation, which requires higher titer of ecdysone [29,44], as confirmed in the process of transition from prepupa to pupa in *D. melanogaster *[24,27]. After pupation, histolysis and histogenesis are occurring intensively and simultaneously, much more of this functional RNA should be required for the double missions, accordingly. The expression profile of *bmo-let-7 *fell at day 7 pupa in female group, and at day 8 in male group, when the ecdysone is also lowly produced [45,46], then resumed to the maximum just before adult emergence corresponding to the rising ecdysone [46]. The expression falling during pupal-adult metamorphosis of both female and male should have occurred synchronously if they had been incubated under the same temperature and humidity.

JH and Ecd function oppositely on the developmental metamorphosis of the silkworm. JH can induce the expression of some genes and repress the expression of other genes activated by Ecd [22,47]. JH is highly secreted by corpus allatum at early period of each larva stage, and Ecd is highly secreted by prothoracic gland at late period of each larva stage [27,48]. Insect metamorphosis is triggered by Ecd in the absence of JH, and is carried out by self-destructive mechanisms of programmed cell death (PCD) [49]. The *let-7 *temporal expression alterations are in synchronization with the pulse of ecdysone [14], as was further confirmed by our northern blotting results (Figs. 6, 7). Large amount of Ecd is secreted in the late fifth instar or in just ripe silkworm in favor of spinning cocoons and molting in pupation [45]. JH is required at late pupa stage to promote the development of ovary and accumulation of yelk (or the maturing of oocytes) and to maintain the functions of testicles. A previous study revealed that in *B.mori*, the prothoracic gland hormone secreted during the first day after larval-pupal ecdysis might be responsible for the development of the ovaries during the pupal period, especially essential for the initiation of ovarian development [49]. After pupation, high level ecdysone is required to drive the tissue differentiation and organ formation of adult moth [44]. Therefore, the Ecd-induced microRNA, *bmo-let-7*, is highly expressed in pupae. However, the mechanism of how *bmo-let-7 *responds to ecdysone and *Broad-Complex *pathways in the silkworm is worthy of further study.

### *bmo-let-7 *is widely expressed in tissues/organs of female and male individuals of the fifth-instar day-3 larvae

**Figure 9:**
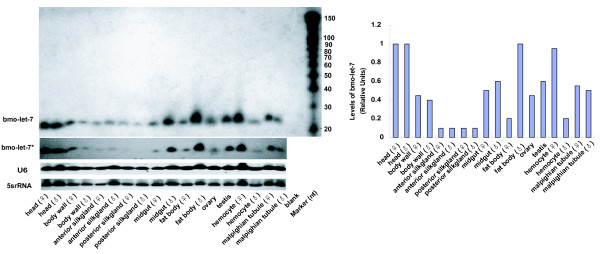
***bmo-let-7 *expression profile in tissues from females and males of fifth-instar day-3 larvae**. Eighteen tissues were harvested from females and males of day-3 5^th ^instar larvae bred at 25°C and 85% H.R. The two antisense probes(anti-bmo-let-7 and anti-bmo-let-7*) were firstly hybridized on different blots, respectively, with equal amount of small RNAs on all loading wells. After stripping, exchanged the probe for the other blot. The candidate mature sequence is 22 nt or 23 nt, and the potential precursor is 72 nt or 87 nt long, but the precursor transcribed by silkworm genome can not be accumulated enough to be seen by Northern blotting. 5srRNA and U6 were used as the loading controls and levels of *bmo-let-7 *RNA are quantified relative to 5srRNA.

*bmo-let-7 *was moderately expressed in female and male individuals at the fifth instar day-3 (Fig. 7B). To correlate expression domains with the functions, we determined the tissue specificity of *bmo-let-7 *expression in various tissues of larvae in day-3 of the fifth instar. Reproducible and consistent results were confirmed by independently parallel Northern blotting experiments with the two antisense probes (see in Methods). The *let-7 *small RNA of 22 nt or 23 nt was present in all tissues studied (Fig. 9). Moderate signals were observed in body wall, midgut, gonad and malpighian tubule, whereas a weak positive signal appeared in anterior silk gland and posterior silk gland, and strong signal was observed in the heads. Sex differences in the expression level were prominent in fat body and hemocyte, however. The female fat body showed weak signal but the male fat body exhibited very strong one. On the contrary, *bmo-let-7 *was highly expressed in the female hemocyte but lowly in male hemocyte. The distinct differences in the expression of *bmo-let-7 *imply that some unknown mechanisms responsible for its transcription might be functioning differently in fat body and hemocyte of female and male silkworm.

Tissue-specific or spatial expression patterns have also been demonstrated in other organisms. As shown in *D. melanogaster*, *let-7 *is widely expressed in a variety of tissues from prepupae, such as brains, imaginal discs, fat bodies, salivary glands, and Malphigian tubules, with relatively higher levels in fat bodies and imaginal discs, and it was also detected in adult ovaraies and carcasses, suggesting that it could regulate diverse metamorphic processes, such as the terminal differentiation of imaginal discs and apoptosis of salivary glands and fat bodies [14]. Furthermore, various tissues from human also express *let-7 *RNA, including brain, heart, kidney, liver, lung, trachea, bone marrow, colon, small intestine, spleen, stomach, and thymus, and the lowest level of human *let-7 *is observed in bone marrow likely due to a large proportion of immature cell in it [8]. Different expression profiles in various tissues from the silkworm indicate that *bmo-let-7 *might not only function in triggering transitions of temporal stages, but more broadly in a variety of metabolisms because each microRNA can control hundreds of target genes [50].

### Expression profile in tissues from just mounting to day-3 pupae suggests its potential roles in histolysis and histogenesis

**Figure 10:**
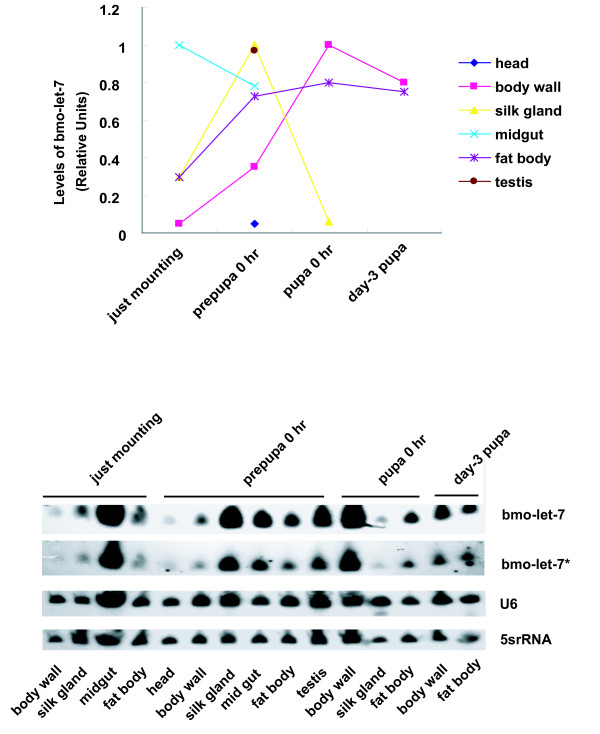
***bmo-let-7 *expression in male tissues from just mounting to day-3 pupa**. At day-3 pupa, it is unlikely to get enough silk gland and midgut, say nothing of small RNAs. Only two tissues were harvested at this period. 5srRNA and U6 were used as the loading controls and levels of *bmo-let-7 *RNA are quantified relative to U6.

Tissues from different stages here presented profiles breadthwise and lengthwise (Fig. 10). The prospective fates of organs and tissues such as head, body wall, silk gland, midgut, fat body and gonad during metamorphic transformation are different. Silk gland and midgut will be completely histolyzed during the pupal stage [51-53], and fat body undergoes reorganization as well as histolysis during larval-pupal transformation [54] and predetermined imaginal tissues differentiate and grow into organs and external structures [53,55]. The histolysis of larval silk gland, fat body and midgut is typical of programmed cell death [49]. Thus, the expression profile of *bmo-let-7 *in tissues from just mounting to day-3 pupae may reflect its association with histolysis and apoptosis.

The individuals at just mounting expressed large amount of *bmo-let-7 *small RNA (Fig. 8A), but the body wall at this period only showed very weak signal (Fig.10). Silk gland and fat body at this time shared a moderate amount. Midgut, however, showed very high level, suggesting that midgut histolysis was triggered once the silkworm stopped eating or just before mounting. When silkworms have finished spinning cocoons three days after mounting, the silk gland accomplishes its mission and undergoes histolysis. At this time, high level expression of *let-7 *was detected in silk gland and testis; moderate level was observed in midgut and fat body, and a weak signal appeared in head and body wall, suggesting that *let-7 *also promotes the apoptosis of silk gland and the maturation of testisis, as demonstrated during the metamorphosis in *D. melanogaster *[14]. Just after pupation (pupa 0 hr), silk gland enters the late stage of histolysis, body wall and fat body are newly reorganized. *let-7 *was very highly expressed in body wall and fat body, but very lowly expressed in silk gland of the newborn pupa, suggesting that *let-7 *functions in the histogenesis of body wall and fat body on the basis of the conclusion that *lin-4 *and *let-7 *control the timing of cell differentiation and proliferation [50,56]. At day-3 pupae, weak expression signals were observed in midgut and silk gland because these two tissues are almost completely histolyzed, but intensive signals appeared in body wall and fat body which are still undergoing reorganization as well as histolysis. Taken together, during the metamorphosis from just mounting to day-3 pupa, the signal intensity in body wall and fat body was rising to the utmost at newborn pupa, then fell at day -3 pupa. The high level expression of *bmo-let-7 *in fat body and other tissues is very likely to be resulted from the ecdysone induction, considering the evidence that the programmed autophagy in the *Drosophila *fat body is induced by ecdysone [57,58]. Silk gland showed the highest expression level just after spinning (prepupa 0 hr), and exhibited a falling signal during the undergoing of apoptosis. In midgut, however, the expression level of *bmo-let-7 *reached the maximum at just mounting, and then decreased. Although *bmo-let-7 *expression is generally response to ecdysone pulse in various tissues as well as in developmental stages, more precise time points and tissues will be investigated to further determine if the expression of *bmo-let-7 *is directly induced by ecdysone in the silkworm.

### Effect of ecdysone change on the expression of *bmo-let-7 *in cultured cells

**Figure 11:**
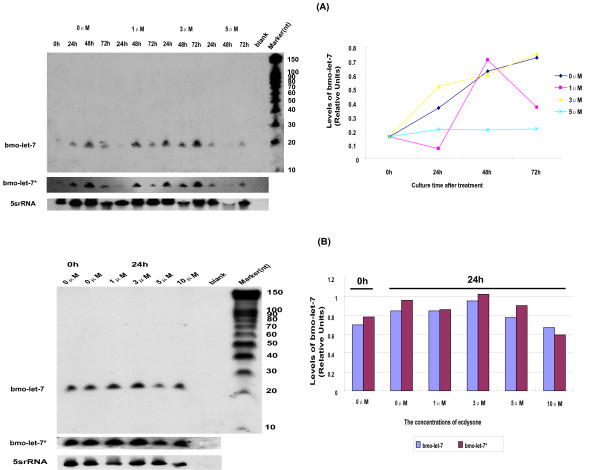
**The effect of concentrations of ecdysone on the expression of *bmo-let-7 *in cultured cells**. (A). The effect of ecdysone for 72 h. (B). The effect of ecdysone for 24 h. The cells were cultured in Grace's medium with or without ecdysone for the periods of time shown. The small RNAs were harvested and two probes for *bmo-let-7 *were used in the detection of Northern blot. 0 h is just before treating with ecdysone or the beginning of treatment with ecdysone, and all cell cultures are synchronous in this assay by transferring from the common vessel. Levels of *bmo-let-7 *are quantified relative to the loading control, 5srRNA.

The expression of *bmo-let-7 *has been systematically investigated in the whole body and tissues or organs during the development of silkworm. The results suggested that *bmo-let-7 *generally responds to the pulse of ecdysone. Cell line culture experiments were then carried out in order to test whether ecdysone is required to sustain the expression of *bmo-let-7 *and how the concentrations of ecdysone are influencing its expression. This small RNA expression trends differed even if it was detectable in all cell samples, which have been incubated in the presence of different levels of ecdysone for different periods (Fig. 11). For the control cells (0 μM ecdysone), *bmo-let-7 *RNA level increased straightly during the whole culture time, with a gentle inflexion at 48 h (Fig. 11A ). When the cells were treated with 3 μM ecdysone, *bmo-let-7 *was initially up-regulated, then reached a plateau shared by the control at 72 h. Lower level of ecdysone, such as 1 μM, seems to exert diverse influence on *bmo-let-7*, down-regulating it slightly before 24 h, up-regulating it distinctly from 24 h to 48 h, then down-regulating it again (Fig. 11A). To avoid the confusion resulted from possible experiment discrepancy, we especially investigated the effects of different levels of ecdysone at 24 h after incubation (Fig. 11B). *bmo-let-7 *was slightly upregulated in the presence of 1 μM ecdysone and most effectively induced by 3 μM ecdysone. However, after treated with 5 μMecdysone, *bmo-let-7 *RNA level was almost always stable during the whole examined span of 72 h, and dramatically decreased in the presence of 10 μM. This suggests that the expression of *bmo-let-7 *might be induced by low-level ecdysone but repressed by high-level ecdysone.

The silkworm ovary itself has been confirmed to express *bmo-let-7 *(Fig.9), and ovarian cell line BmN-SWU1 was also validated to produce this small RNA even if no ecdysone was added into the culture medium (Fig.11). During the culture periods, the untreated cells are very likely to produce ecdysone, so we cannot rule out the possibility that the endogenetic and adscititious ecdysones are functioning together in inducing or repressing the expression of *bmo-let-7 *RNA. Since *bmo-let-7 *is undetectable during the development of silkworm's embryo (Figs. 6, 7), the embryo cell line is also unlikely to produce detectable *bmo-let-7 *RNA. It would be interesting to look at whether the expression of *bmo-let-7 *RNA could be observed when the embryo cell line would have been incubated for certain length of time in the presence of ecdysone. The tissue-specificity and the timing of *let-7 *expression are conserved in invertebrates, suggesting that it could exert widely in mediating diverse metamorphic processes [8]. The programmed ecdysis behaviour in larval, pupal and adult stages occurs in all of the holometabolous insects, and depends on coordinated expression of a series of genes related to peptide signaling [59]. Very low-level expression of *let-7 *can even be observed in the *npr*^6 ^mutants of *Drosophila*, which are unresponsive to the ecdysone pulse at the end of the last larval stage (L3) due to the lack of four *BR-C *isoforms [14]. It could be speculated that ecdysone might be only one of the factors regulating *bmo-let-7*, and some higher upstream components of the heterochronic pathway could also contribute to its expression.

## Conclusion

A new candidate member (*bmo-let-7*) of *let-7 *family in the silkworm (*B. mori*) was identified by computational approach and its expression was profiled by Northern blotting. Lines of evidence confirmed that *bmo-let-7 *is temporally and spatially expressed in the silkworm and the expression is in response to the pulse of ecdysone, and might be related to a number of biological processes such as cell proliferation, histogenesis, histolysis, organogenesis and apoptosis. It is shown from the cell culture experiment that induction or repression effects of ecdysone on the expression of *bmo-let-7 *are concentration-dependent.

## Methods

### Computational analyses

Both sequence homology search and miRscan program were used to identify the orthologs of the *let-7 *gene in the silkworm genome as reported [60,61]. The mature *let-7 *sequences of *C. elegans *and *D. melanogaster *were downloaded from the miRbase [62], and were used as query sequences to BLASTN against the silkworm genome in NCBI with default parameters and a non-stringent cutoff of *E *> 1.8 [60]. Then, the miRscan program [63] was used to scan the genome of silkworm as reported in other organisms [60,64]. As a result, over 100 candidate microRNA genes were found (data not shown). The precursor's stem-loop structure was predicted using mfold version 3.2 [65]. All known microRNAs and their precursors were downloaded from the miRbase [62]. Ninety mature sequences of this family were submitted to logo analysis by using the WebLogo program [66]. Twenty-six selected *let-7 *members and the sequences of their precursors were aligned into phylogenetic tree by using MEGA v3.0 [67]. The alignments were processed by using of Boxshade [68].

### Animal breeding and sample preparation

Female moths of the domesticated silkworm (*Bombyx mori*), DaZao, were allowed to lay eggs for 4 hr at 25°C and the developing eggs were incubated at 25°C from the oviposition until hatching, the first day being the day of oviposition. When developing eggs were incubated at 25°C, head pigmentation came on day 7 (6 × 24 hr after oviposition), body pigmentation appeared on day 9 (8 × 24 hr after oviposition), and more than 90% of the eggs hatched on the tenth day. To prevent entering the diapause, the fertilized eggs were treated with a hydrochloric acid solution, from 15 to 20 hr after oviposition at 25°C. After hatching, larvae of the silkworm were reared on mulberry leaves under a 12-h light/12-h dark photoperiod at 25°C and 85 % H.R. and harvested at desired developmental stages. In order to obtain populations of *B. mori *at various larval stages, animals were synchronized after oviposition by means of cold storage and acid treatment, keeping the diapause eggs at 4°C for at least three months followed by 5 min of acid treatment with a hydrochloric acid solution at 46°C. Moreover, developmental landmarks, including hatching, larval molting, mounting, spinning, pupariation, and eclosion, were used for more precise staging. Each organ or tissue was separated by manual dissection. The mulberry residues were completely removed from the midgut and other tissues by rinsing with DEPC-treated water.

Firstly, a general temporal expression profile was revealed using 15 stage-specific samples across the whole life cycle of silkworm from embryo to imago (adult silkworm). Unfertilized egg (ova) or pre-laid egg, as the contrast of oosperm, was collected from the unmated neonatal female moth. The oviduct fastening the unfertilized egg was unremoved or removed for further confirmation. The sex differences were taken into account from day-3 of the fifth instar to the adult moth. In order to describe the expression pattern elaborately, we then shortened the sample-harvested intervals. Fifteen and fourteen samples were taken for the metamorphosis of female and male pupae, respectively. The whole life cycle was divided into three parts for convenience of blotting, from embryo to the third molt, from the fourth instar to day-7 of the fifth instar and from spinning (just mounting) to day 2 of imago. To know the transverse expression profile in various tissues, nine kinds of tissues or organs of day-3 of the fifth instar were harvested from female and male silkworms, respectively. And different tissues from just mounting to pupa stages were also taken to present a lengthways expression profile.

### Cell culture and ecdysone treatment

A new cell line of *Bombyx mori*, BmN-SWU1, was used to examine the effect on *bmo-let-7 *caused by different concentrations of ecdysone. The BmN-SWU1 cell line was recently established in our lab by using of ovary tissue with different differentiated types, and the cells were cultured in Grace's medium supplemented with 10% fetal bovine serum at 25°C. Ecdysone treatment was as follows: cells were transferred from a common big culture flask and then plated in 25-cm^2 ^flasks containing 3 ml of medium and allowed to grow for 48 h when they reach about 80% of confluence. 20-hydroxyecdysone (Sigma) was then added to a final gradient concentrations of 1 μM, 3 μM, 5 μM and 10 μM, respectively. Ecdysone-untreated cells were used as the control. The cells cultures, with or without ecdysone, were timed synchronously from the beginning of incubation to various lengths of time (0–72 h). All samples cells were harvested every 24 h.

### Small RNA isolation and Northern blot analysis

We used the small RNA of the mulberry leaves as a control to eliminate the possible contamination. All samples prepared were frozen in liquid nitrogen before use. Small RNAs were isolated with *mir*Vana™ miRNA Isolation Kit (Ambion) according to the Instruction Manual. The extracted small RNAs were quantified by spectrophotometer, Gene Quant (Bio-Rad) before loading. Blots were prepared by electrophoresing 15 μg small-sized RNAs per lane on a denaturing 12% polyacryamide-7 mol/l urea gel at 200 V for 1 hr, then at 300 V for 2 h, followed by electroblotting to positively charged nylon membranes (Ambion) by using semi-dry Trans-Blot Electrophoretic Transfer (Bio-Rad). After electroblotting, the RNAs were fixed to the membrane by UV cross-linking (1200 μJ, Stratalinker; Stratagene) followed by baking in a vacuum oven at 80°C for 30 min. DNA oligonucleotides complementary to predicted candidate *bmo-let-7*, *cel-lin-4*, U6 RNA and 5srRNA were synthesized (Sangon, Shanghai). Two antisense probes were used to confirm the reproducible and consistent results. The 5'-ends of the DNA and the Decade Markers (Ambion) were labeled with [γ-^32^P] ATP (Amersham) using T4 polynucleotide kinase (Takara) and submitted to purification by using Purification Cartridge (Ambion). The membrane was pre-hybridized in prehybridization solution containing 6 × SSC, 10 × Denhardt's solution, 0.2% SDS and 300 μg salmon sperm DNA (Ambion) at 50°C for about 10 hr. Then membranes were hybridized in hybridization solution containing 6 × SSC, 5 × Denhardt's solution, 0.2% SDS and 300 μg denatured sheared salmon sperm DNA (Ambion) with 1–5 × 10^6 ^cpm eluted radiolabeled oligo probes at temperature of 10–15°C below the calculated dissociation temperature (Td) for at least 10 h. The blots were washed thrice for 5 min each at 37°C with 6 × SSC and 0.2% SDS and once at 42°C for 30 min. After the final wash, wrap the blot in plastic wrap and expose to X-ray film at -70°C for proper time. Strip off the former probe for reprobing by washing at 90°C in 0.1 × SSC, 0.5% SDS. Radioactive signals were quantified with ImageQuant software package (Molecular Dynamics). The relative levels of *let-7 *transcript were presented as the ratio of let-7 and U6 or 5srRNA radioactive signals normalized to a 0~1 scale.

### The 5' kinase labeled oligo probes

To confirm the reproducible and consistent results, parallel tests were conducted using probes of *bmo-let-7 *and bmo-let-7*, one of which with only an additional nucleotide at the 3'end. The antisence and sense probes of *bmo-let-7*, bmo-let-7*and *cel-lin-4 *were used to investigate if the functional small RNA is a single strand. U6 RNA and 5srRNA were used as the loading controls.

Sequences from 5' to 3' ends:

*bmo-let-7 *antisense, TACTATACAACCTACTACCTCA;

*bmo-let-7 *sense, TGAGGTAGTAGGTTGTATAGTA;

bmo-let-7* antisense, TACTATACAACCTACTACCTCAA;

bmo-let-7* sense, TTGAGGTAGTAGGTTGTATAGTA;

*cel-lin-4 *antisense, TCACACTTGAGGTCTCAGGGA

*cel-lin-4 *sense, TCCCTGAGACCTCAAGTGTGA

U6 antisense, GCAGGGGCCATGCTAATCGTCTCTGTATCG;

5srRNA antisense, GTACTGACCACGCCCGATGTTGCTTGACTT

## Authors' contributions

Shiping-Liu conceived the study, reared and harvested all the samples, carried out the Northern blotting experiments, performed the computational prediction and drafted the manuscript. Qingyou-Xia, Ping Zhao participated in conceiving the study and helped to draft the manuscript. Tingcai-Cheng participated in the design of the study and carried out the sequence alignment. Kaili Hong cultured the cells and performed ecdysone treatment. Zhonghuai-Xiang conceived and coordinated the study. All authors read, modified and approved the final manuscript.
